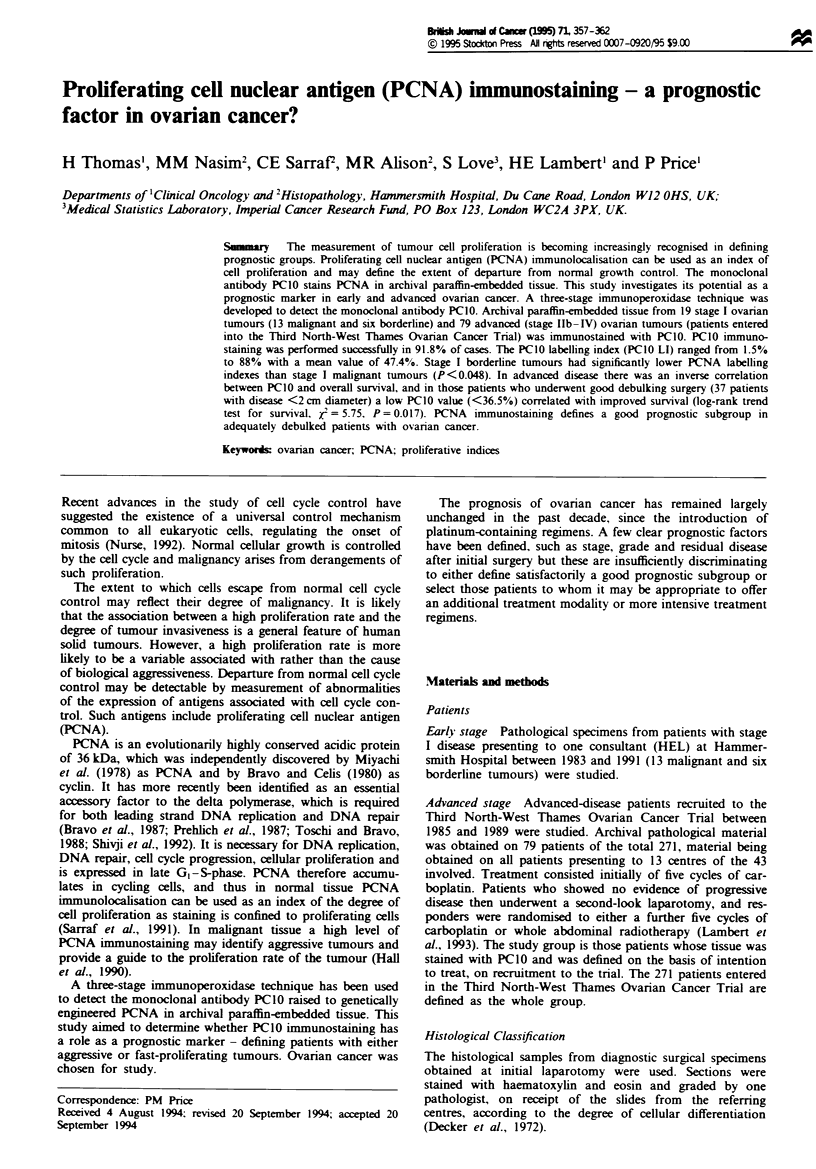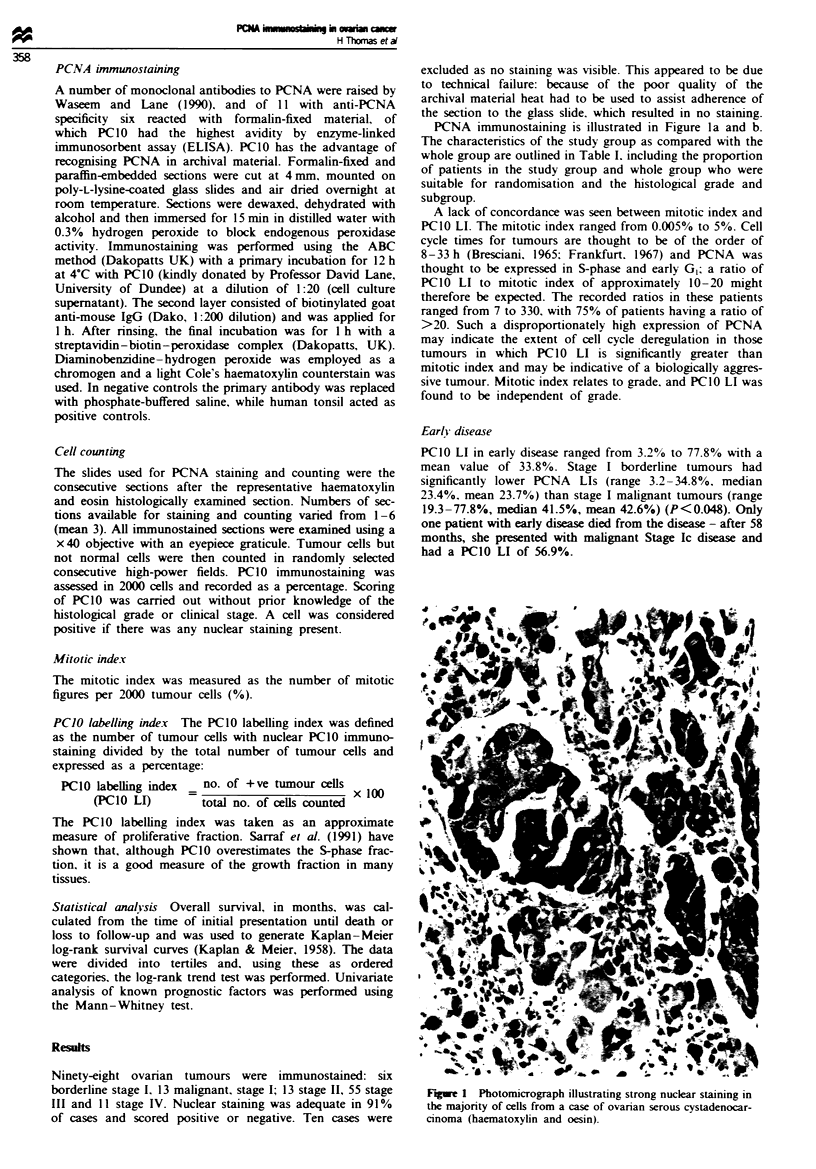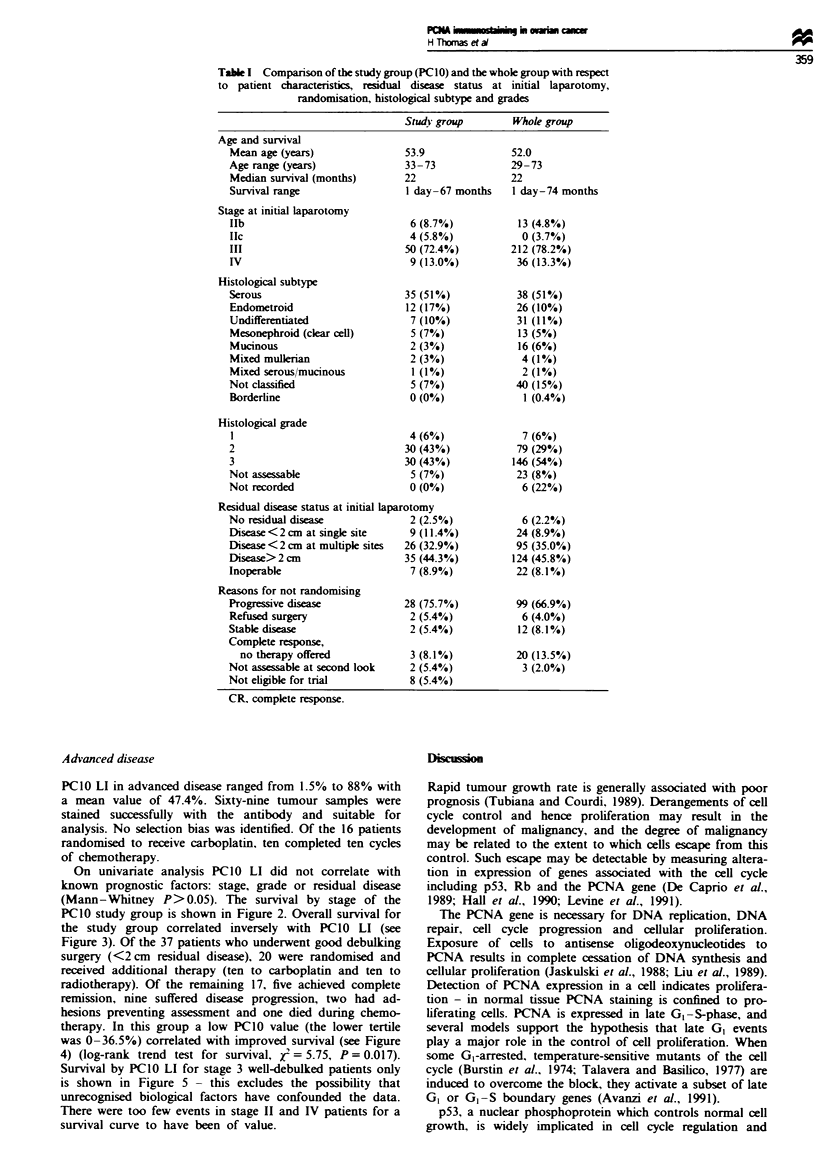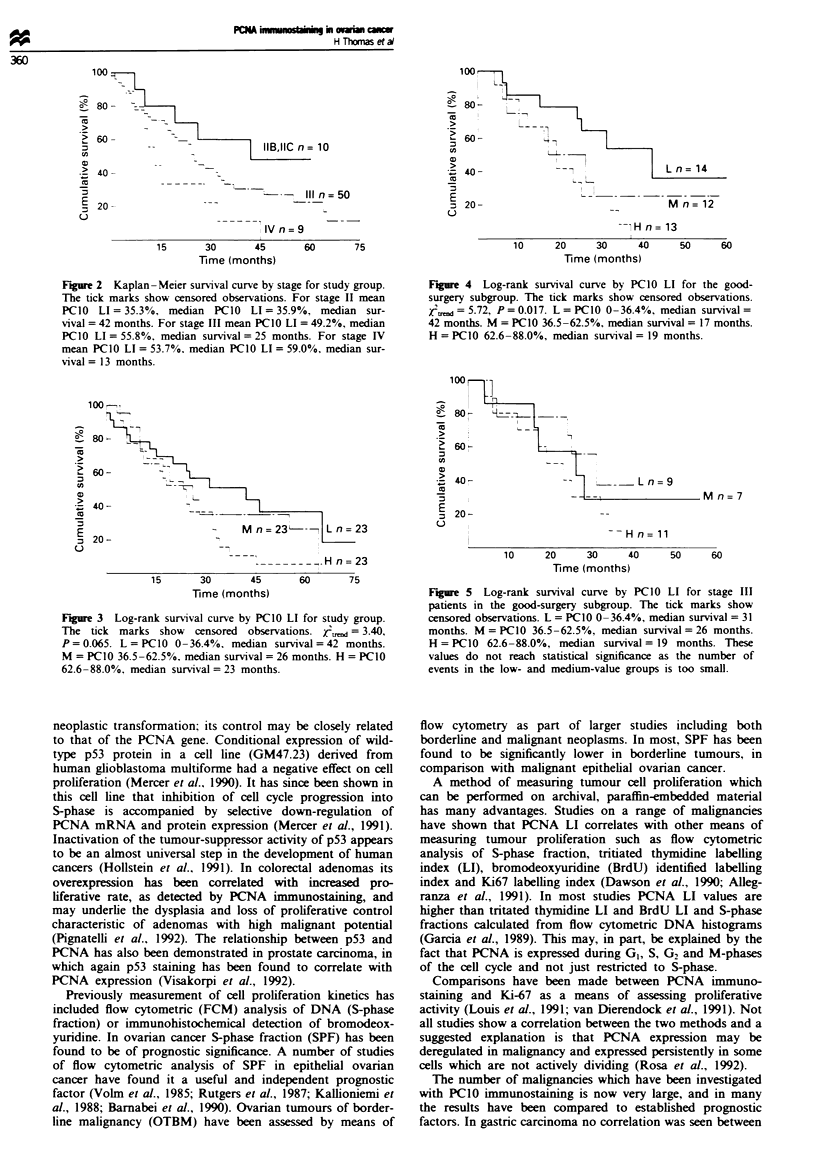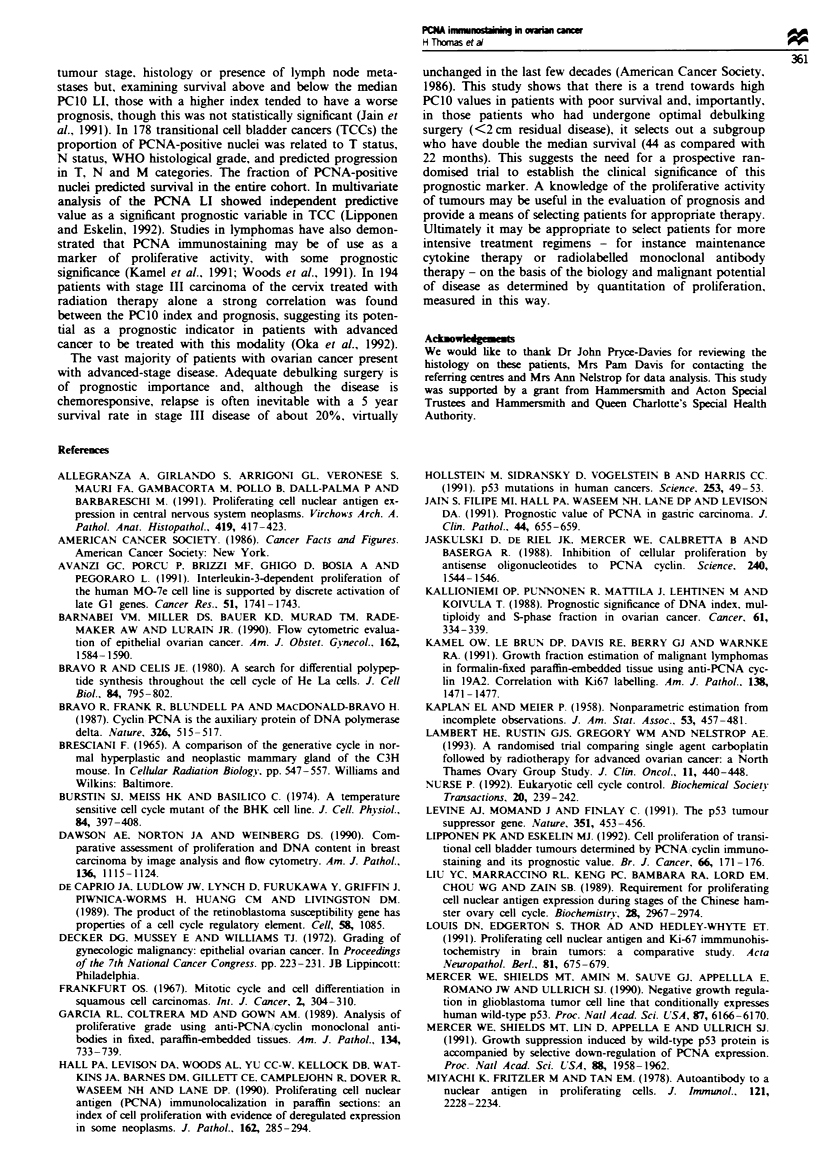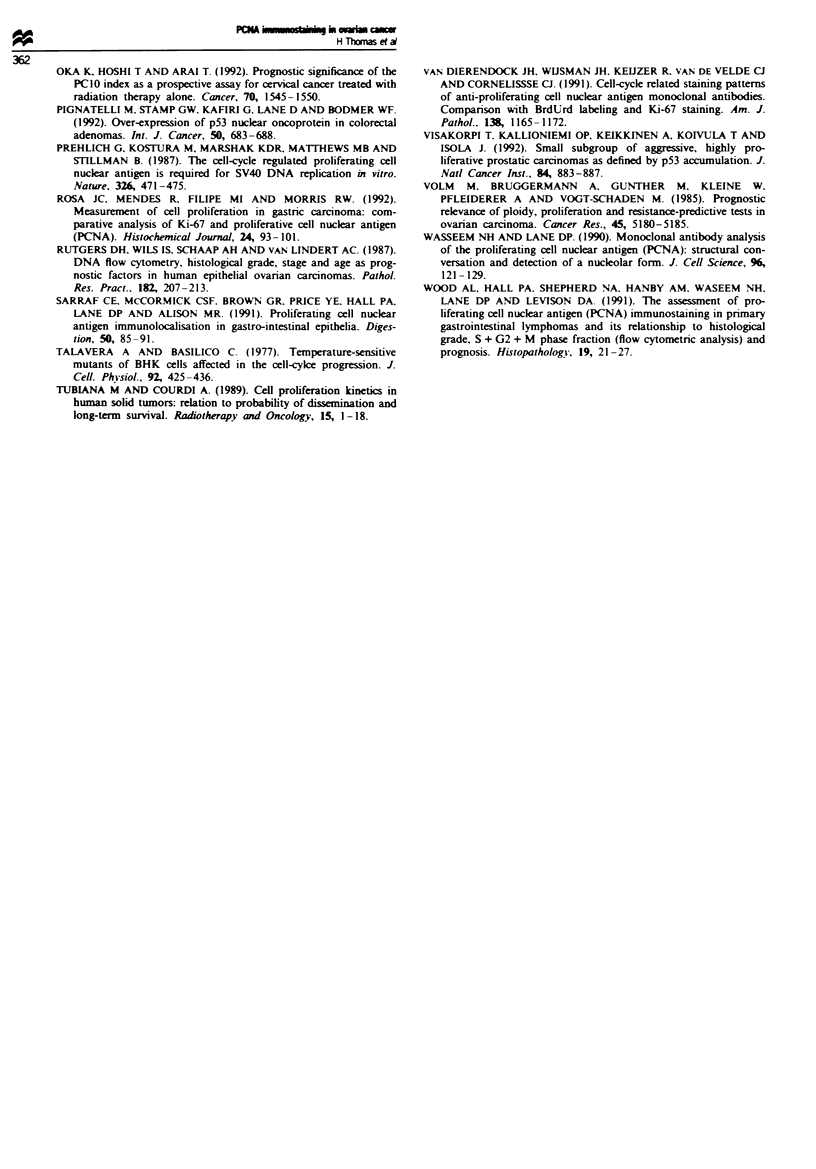# Proliferating cell nuclear antigen (PCNA) immunostaining--a prognostic factor in ovarian cancer?

**DOI:** 10.1038/bjc.1995.72

**Published:** 1995-02

**Authors:** H. Thomas, M. M. Nasim, C. E. Sarraf, M. R. Alison, S. Love, H. E. Lambert, P. Price

**Affiliations:** Department of Clinical Oncology, Hammersmith Hospital, UK.

## Abstract

**Images:**


British Journal of Cancer (1995) 71, 357-362

© 1995 Stockton Press All rights reserved 0007-0920/95 $9.00

### Proliferating cell nuclear antigen (PCNA) immunostaining – a prognostic factor in ovarian cancer?

H Thomas[1], MM Nasim[2], CE Sarraf[2], MR Alison[2], S Love[3], HE Lambert[1] and P Price[1]

*Departments of [1]Clinical Oncology and [2]Histopathology, Hammersmith Hospital, Du Cane Road, London W12 0HS, UK; [3]Medical Statistics Laboratory, Imperial Cancer Research Fund, PO Box 123, London WC2A 3PX, UK.*

**Summary** The measurement of tumour cell proliferation is becoming increasingly recognised in defining prognostic groups. Proliferating cell nuclear antigen (PCNA) immunolocalisation can be used as an index of cell proliferation and may define the extent of departure from normal growth control. The monoclonal antibody PC10 stains PCNA in archival paraffin-embedded tissue. This study investigates its potential as a prognostic marker in early and advanced ovarian cancer. A three-stage immunoperoxidase technique was developed to detect the monoclonal antibody PC10. Archival paraffin-embedded tissue from 19 stage I ovarian tumours (13 malignant and six borderline) and 79 advanced (stage IIb-IV) ovarian tumours (patients entered into the Third North-West Thames Ovarian Cancer Trial) was immunostained with PC10. PC10 immunostaining was performed successfully in 91.8% of cases. The PC10 labelling index (PC10 LI) ranged from 1.5% to 88% with a mean value of 47.4%. Stage I borderline tumours had significantly lower PCNA labelling indexes than stage I malignant tumours ($P<0.048$). In advanced disease there was an inverse correlation between PC10 and overall survival, and in those patients who underwent good debulking surgery (37 patients with disease <2 cm diameter) a low PC10 value (<36.5%) correlated with improved survival (log-rank trend test for survival, $\chi^2 = 5.75$, $P = 0.017$). PCNA immunostaining defines a good prognostic subgroup in adequately debulked patients with ovarian cancer.

**Keywords:** ovarian cancer; PCNA; proliferative indices

Recent advances in the study of cell cycle control have suggested the existence of a universal control mechanism common to all eukaryotic cells, regulating the onset of mitosis (Nurse, 1992). Normal cellular growth is controlled by the cell cycle and malignancy arises from derangements of such proliferation.

The extent to which cells escape from normal cell cycle control may reflect their degree of malignancy. It is likely that the association between a high proliferation rate and the degree of tumour invasiveness is a general feature of human solid tumours. However, a high proliferation rate is more likely to be a variable associated with rather than the cause of biological aggressiveness. Departure from normal cell cycle control may be detectable by measurement of abnormalities of the expression of antigens associated with cell cycle control. Such antigens include proliferating cell nuclear antigen (PCNA).

PCNA is an evolutionarily highly conserved acidic protein of 36 kDa, which was independently discovered by Miyachi *et al.* (1978) as PCNA and by Bravo and Celis (1980) as cyclin. It has more recently been identified as an essential accessory factor to the delta polymerase, which is required for both leading strand DNA replication and DNA repair (Bravo *et al.*, 1987; Prehlich *et al.*, 1987; Toschi and Bravo, 1988; Shivji *et al.*, 1992). It is necessary for DNA replication, DNA repair, cell cycle progression, cellular proliferation and is expressed in late $G_1$-S-phase. PCNA therefore accumulates in cycling cells, and thus in normal tissue PCNA immunolocalisation can be used as an index of the degree of cell proliferation as staining is confined to proliferating cells (Sarraf *et al.*, 1991). In malignant tissue a high level of PCNA immunostaining may identify aggressive tumours and provide a guide to the proliferation rate of the tumour (Hall *et al.*, 1990).

A three-stage immunoperoxidase technique has been used to detect the monoclonal antibody PC10 raised to genetically engineered PCNA in archival paraffin-embedded tissue. This study aimed to determine whether PC10 immunostaining has a role as a prognostic marker – defining patients with either aggressive or fast-proliferating tumours. Ovarian cancer was chosen for study.

Correspondence: PM Price
Received 4 August 1994; revised 20 September 1994; accepted 20 September 1994

The prognosis of ovarian cancer has remained largely unchanged in the past decade, since the introduction of platinum-containing regimens. A few clear prognostic factors have been defined, such as stage, grade and residual disease after initial surgery but these are insufficiently discriminating to either define satisfactorily a good prognostic subgroup or select those patients to whom it may be appropriate to offer an additional treatment modality or more intensive treatment regimens.

### Materials and methods

### *Patients*

*Early stage* Pathological specimens from patients with stage I disease presenting to one consultant (HEL) at Hammersmith Hospital between 1983 and 1991 (13 malignant and six borderline tumours) were studied.

*Advanced stage* Advanced-disease patients recruited to the Third North-West Thames Ovarian Cancer Trial between 1985 and 1989 were studied. Archival pathological material was obtained on 79 patients of the total 271, material being obtained on all patients presenting to 13 centres of the 43 involved. Treatment consisted initially of five cycles of carboplatin. Patients who showed no evidence of progressive disease then underwent a second-look laparotomy, and responders were randomised to either a further five cycles of carboplatin or whole abdominal radiotherapy (Lambert *et al.*, 1993). The study group is those patients whose tissue was stained with PC10 and was defined on the basis of intention to treat, on recruitment to the trial. The 271 patients entered in the Third North-West Thames Ovarian Cancer Trial are defined as the whole group.

### *Histological Classification*

The histological samples from diagnostic surgical specimens obtained at initial laparotomy were used. Sections were stained with haematoxylin and eosin and graded by one pathologist, on receipt of the slides from the referring centres, according to the degree of cellular differentiation (Decker *et al.*, 1972).

*PCNA immunostaining*

A number of monoclonal antibodies to PCNA were raised by Waseem and Lane (1990), and of 11 with anti-PCNA specificity six reacted with formalin-fixed material, of which PC10 had the highest avidity by enzyme-linked immunosorbent assay (ELISA). PC10 has the advantage of recognising PCNA in archival material. Formalin-fixed and paraffin-embedded sections were cut at 4 mm, mounted on poly-L-lysine-coated glass slides and air dried overnight at room temperature. Sections were dewaxed, dehydrated with alcohol and then immersed for 15 min in distilled water with 0.3% hydrogen peroxide to block endogenous peroxidase activity. Immunostaining was performed using the ABC method (Dakopatts UK) with a primary incubation for 12 h at 4°C with PC10 (kindly donated by Professor David Lane, University of Dundee) at a dilution of 1:20 (cell culture supernatant). The second layer consisted of biotinylated goat anti-mouse IgG (Dako, 1:200 dilution) and was applied for 1 h. After rinsing, the final incubation was for 1 h with a streptavidin-biotin-peroxidase complex (Dakopatts, UK). Diaminobenzidine-hydrogen peroxide was employed as a chromogen and a light Cole's haematoxylin counterstain was used. In negative controls the primary antibody was replaced with phosphate-buffered saline, while human tonsil acted as positive controls.

*Cell counting*

The slides used for PCNA staining and counting were the consecutive sections after the representative haematoxylin and eosin histologically examined section. Numbers of sections available for staining and counting varied from 1-6 (mean 3). All immunostained sections were examined using a ×40 objective with an eyepiece graticule. Tumour cells but not normal cells were then counted in randomly selected consecutive high-power fields. PC10 immunostaining was assessed in 2000 cells and recorded as a percentage. Scoring of PC10 was carried out without prior knowledge of the histological grade or clinical stage. A cell was considered positive if there was any nuclear staining present.

*Mitotic index*

The mitotic index was measured as the number of mitotic figures per 2000 tumour cells (%).

*PC10 labelling index* The PC10 labelling index was defined as the number of tumour cells with nuclear PC10 immunostaining divided by the total number of tumour cells and expressed as a percentage:

$$\text{PC10 labelling index (PC10 LI)} = \frac{\text{no. of +ve tumour cells}}{\text{total no. of cells counted}} \times 100$$

The PC10 labelling index was taken as an approximate measure of proliferative fraction. Sarraf *et al.* (1991) have shown that, although PC10 overestimates the S-phase fraction, it is a good measure of the growth fraction in many tissues.

*Statistical analysis* Overall survival, in months, was calculated from the time of initial presentation until death or loss to follow-up and was used to generate Kaplan-Meier log-rank survival curves (Kaplan & Meier, 1958). The data were divided into tertiles and, using these as ordered categories, the log-rank trend test was performed. Univariate analysis of known prognostic factors was performed using the Mann-Whitney test.

### Results

Ninety-eight ovarian tumours were immunostained: six borderline stage I, 13 malignant, stage I; 13 stage II, 55 stage III and 11 stage IV. Nuclear staining was adequate in 91% of cases and scored positive or negative. Ten cases were excluded as no staining was visible. This appeared to be due to technical failure: because of the poor quality of the archival material heat had to be used to assist adherence of the section to the glass slide, which resulted in no staining.

PCNA immunostaining is illustrated in Figure 1a and b. The characteristics of the study group as compared with the whole group are outlined in Table I, including the proportion of patients in the study group and whole group who were suitable for randomisation and the histological grade and subgroup.

A lack of concordance was seen between mitotic index and PC10 LI. The mitotic index ranged from 0.005% to 5%. Cell cycle times for tumours are thought to be of the order of 8-33 h (Bresciani, 1965; Frankfurt, 1967) and PCNA was thought to be expressed in S-phase and early $G_1$; a ratio of PC10 LI to mitotic index of approximately 10-20 might therefore be expected. The recorded ratios in these patients ranged from 7 to 330, with 75% of patients having a ratio of >20. Such a disproportionately high expression of PCNA may indicate the extent of cell cycle deregulation in those tumours in which PC10 LI is significantly greater than mitotic index and may be indicative of a biologically aggressive tumour. Mitotic index relates to grade, and PC10 LI was found to be independent of grade.

*Early disease*

PC10 LI in early disease ranged from 3.2% to 77.8% with a mean value of 33.8%. Stage I borderline tumours had significantly lower PCNA LIs (range 3.2-34.8%, median 23.4%, mean 23.7%) than stage I malignant tumours (range 19.3-77.8%, median 41.5%, mean 42.6%) ($P<0.048$). Only one patient with early disease died from the disease - after 58 months, she presented with malignant Stage Ic disease and had a PC10 LI of 56.9%.

**Figure 1** Photomicrograph illustrating strong nuclear staining in the majority of cells from a case of ovarian serous cystadenocarcinoma (haematoxylin and oesin).

**Table I** Comparison of the study group (PC10) and the whole group with respect to patient characteristics, residual disease status at initial laparotomy, randomisation, histological subtype and grades

| | *Study group* | *Whole group* |
|---|---|---|
| Age and survival | | |
| Mean age (years) | 53.9 | 52.0 |
| Age range (years) | 33-73 | 29-73 |
| Median survival (months) | 22 | 22 |
| Survival range | 1 day-67 months | 1 day-74 months |
| Stage at initial laparotomy | | |
| IIb | 6 (8.7%) | 13 (4.8%) |
| IIc | 4 (5.8%) | 0 (3.7%) |
| III | 50 (72.4%) | 212 (78.2%) |
| IV | 9 (13.0%) | 36 (13.3%) |
| Histological subtype | | |
| Serous | 35 (51%) | 38 (51%) |
| Endometroid | 12 (17%) | 26 (10%) |
| Undifferentiated | 7 (10%) | 31 (11%) |
| Mesonephroid (clear cell) | 5 (7%) | 13 (5%) |
| Mucinous | 2 (3%) | 16 (6%) |
| Mixed mullerian | 2 (3%) | 4 (1%) |
| Mixed serous/mucinous | 1 (1%) | 2 (1%) |
| Not classified | 5 (7%) | 40 (15%) |
| Borderline | 0 (0%) | 1 (0.4%) |
| Histological grade | | |
| 1 | 4 (6%) | 7 (6%) |
| 2 | 30 (43%) | 79 (29%) |
| 3 | 30 (43%) | 146 (54%) |
| Not assessable | 5 (7%) | 23 (8%) |
| Not recorded | 0 (0%) | 6 (22%) |
| Residual disease status at initial laparotomy | | |
| No residual disease | 2 (2.5%) | 6 (2.2%) |
| Disease < 2 cm at single site | 9 (11.4%) | 24 (8.9%) |
| Disease < 2 cm at multiple sites | 26 (32.9%) | 95 (35.0%) |
| Disease > 2 cm | 35 (44.3%) | 124 (45.8%) |
| Inoperable | 7 (8.9%) | 22 (8.1%) |
| Reasons for not randomising | | |
| Progressive disease | 28 (75.7%) | 99 (66.9%) |
| Refused surgery | 2 (5.4%) | 6 (4.0%) |
| Stable disease | 2 (5.4%) | 12 (8.1%) |
| Complete response, no therapy offered | 3 (8.1%) | 20 (13.5%) |
| Not assessable at second look | 2 (5.4%) | 3 (2.0%) |
| Not eligible for trial | 8 (5.4%) | |

CR, complete response.

### *Advanced disease*

PC10 LI in advanced disease ranged from 1.5% to 88% with a mean value of 47.4%. Sixty-nine tumour samples were stained successfully with the antibody and suitable for analysis. No selection bias was identified. Of the 16 patients randomised to receive carboplatin, ten completed ten cycles of chemotherapy.

On univariate analysis PC10 LI did not correlate with known prognostic factors: stage, grade or residual disease (Mann-Whitney $P > 0.05$). The survival by stage of the PC10 study group is shown in Figure 2. Overall survival for the study group correlated inversely with PC10 LI (see Figure 3). Of the 37 patients who underwent good debulking surgery (<2 cm residual disease), 20 were randomised and received additional therapy (ten to carboplatin and ten to radiotherapy). Of the remaining 17, five achieved complete remission, nine suffered disease progression, two had adhesions preventing assessment and one died during chemotherapy. In this group a low PC10 value (the lower tertile was 0-36.5%) correlated with improved survival (see Figure 4) (log-rank trend test for survival, $\chi^2 = 5.75$, $P = 0.017$). Survival by PC10 LI for stage 3 well-debulked patients only is shown in Figure 5 - this excludes the possibility that unrecognised biological factors have confounded the data. There were too few events in stage II and IV patients for a survival curve to have been of value.

### Discussion

Rapid tumour growth rate is generally associated with poor prognosis (Tubiana and Courdi, 1989). Derangements of cell cycle control and hence proliferation may result in the development of malignancy, and the degree of malignancy may be related to the extent to which cells escape from this control. Such escape may be detectable by measuring alteration in expression of genes associated with the cell cycle including p53, Rb and the PCNA gene (De Caprio *et al.*, 1989; Hall *et al.*, 1990; Levine *et al.*, 1991).

The PCNA gene is necessary for DNA replication, DNA repair, cell cycle progression and cellular proliferation. Exposure of cells to antisense oligodeoxynucleotides to PCNA results in complete cessation of DNA synthesis and cellular proliferation (Jaskulski *et al.*, 1988; Liu *et al.*, 1989). Detection of PCNA expression in a cell indicates proliferation - in normal tissue PCNA staining is confined to proliferating cells. PCNA is expressed in late $G_1$-S-phase, and several models support the hypothesis that late $G_1$ events play a major role in the control of cell proliferation. When some $G_1$-arrested, temperature-sensitive mutants of the cell cycle (Burstin *et al.*, 1974; Talavera and Basilico, 1977) are induced to overcome the block, they activate a subset of late $G_1$ or $G_1$-S boundary genes (Avanzi *et al.*, 1991).

p53, a nuclear phosphoprotein which controls normal cell growth, is widely implicated in cell cycle regulation and

**Figure 2** Kaplan–Meier survival curve by stage for study group. The tick marks show censored observations. For stage II mean PC10 LI = 35.3%, median PC10 LI = 35.9%, median survival = 42 months. For stage III mean PC10 LI = 49.2%, median PC10 LI = 55.8%, median survival = 25 months. For stage IV mean PC10 LI = 53.7%, median PC10 LI = 59.0%, median survival = 13 months.

**Figure 3** Log-rank survival curve by PC10 LI for study group. The tick marks show censored observations. $\chi^2_{trend} = 3.40$, $P = 0.065$. L = PC10 0–36.4%, median survival = 42 months. M = PC10 36.5–62.5%, median survival = 26 months. H = PC10 62.6–88.0%, median survival = 23 months.

**Figure 4** Log-rank survival curve by PC10 LI for the good-surgery subgroup. The tick marks show censored observations. $\chi^2_{trend} = 5.72$, $P = 0.017$. L = PC10 0–36.4%, median survival = 42 months. M = PC10 36.5–62.5%, median survival = 17 months. H = PC10 62.6–88.0%, median survival = 19 months.

**Figure 5** Log-rank survival curve by PC10 LI for stage III patients in the good-surgery subgroup. The tick marks show censored observations. L = PC10 0–36.4%, median survival = 31 months. M = PC10 36.5–62.5%, median survival = 26 months. H = PC10 62.6–88.0%, median survival = 19 months. These values do not reach statistical significance as the number of events in the low- and medium-value groups is too small.

neoplastic transformation; its control may be closely related to that of the PCNA gene. Conditional expression of wild-type p53 protein in a cell line (GM47.23) derived from human glioblastoma multiforme had a negative effect on cell proliferation (Mercer *et al.*, 1990). It has since been shown in this cell line that inhibition of cell cycle progression into S-phase is accompanied by selective down-regulation of PCNA mRNA and protein expression (Mercer *et al.*, 1991). Inactivation of the tumour-suppressor activity of p53 appears to be an almost universal step in the development of human cancers (Hollstein *et al.*, 1991). In colorectal adenomas its overexpression has been correlated with increased proliferative rate, as detected by PCNA immunostaining, and may underlie the dysplasia and loss of proliferative control characteristic of adenomas with high malignant potential (Pignatelli *et al.*, 1992). The relationship between p53 and PCNA has also been demonstrated in prostate carcinoma, in which again p53 staining has been found to correlate with PCNA expression (Visakorpi *et al.*, 1992).

Previously measurement of cell proliferation kinetics has included flow cytometric (FCM) analysis of DNA (S-phase fraction) or immunohistochemical detection of bromodeoxyuridine. In ovarian cancer S-phase fraction (SPF) has been found to be of prognostic significance. A number of studies of flow cytometric analysis of SPF in epithelial ovarian cancer have found it a useful and independent prognostic factor (Volm *et al.*, 1985; Rutgers *et al.*, 1987; Kallioniemi *et al.*, 1988; Barnabei *et al.*, 1990). Ovarian tumours of borderline malignancy (OTBM) have been assessed by means of flow cytometry as part of larger studies including both borderline and malignant neoplasms. In most, SPF has been found to be significantly lower in borderline tumours, in comparison with malignant epithelial ovarian cancer.

A method of measuring tumour cell proliferation which can be performed on archival, paraffin-embedded material has many advantages. Studies on a range of malignancies have shown that PCNA LI correlates with other means of measuring tumour proliferation such as flow cytometric analysis of S-phase fraction, tritiated thymidine labelling index (LI), bromodeoxyuridine (BrdU) identified labelling index and Ki67 labelling index (Dawson *et al.*, 1990; Allegranza *et al.*, 1991). In most studies PCNA LI values are higher than tritated thymidine LI and BrdU LI and S-phase fractions calculated from flow cytometric DNA histograms (Garcia *et al.*, 1989). This may, in part, be explained by the fact that PCNA is expressed during $G_1$, S, $G_2$ and M-phases of the cell cycle and not just restricted to S-phase.

Comparisons have been made between PCNA immunostaining and Ki-67 as a means of assessing proliferative activity (Louis *et al.*, 1991; van Dierendock *et al.*, 1991). Not all studies show a correlation between the two methods and a suggested explanation is that PCNA expression may be deregulated in malignancy and expressed persistently in some cells which are not actively dividing (Rosa *et al.*, 1992).

The number of malignancies which have been investigated with PC10 immunostaining is now very large, and in many the results have been compared to established prognostic factors. In gastric carcinoma no correlation was seen between tumour stage, histology or presence of lymph node metastases but, examining survival above and below the median PC10 LI, those with a higher index tended to have a worse prognosis, though this was not statistically significant (Jain *et al.*, 1991). In 178 transitional cell bladder cancers (TCCs) the proportion of PCNA-positive nuclei was related to T status, N status, WHO histological grade, and predicted progression in T, N and M categories. The fraction of PCNA-positive nuclei predicted survival in the entire cohort. In multivariate analysis of the PCNA LI showed independent predictive value as a significant prognostic variable in TCC (Lipponen and Eskelin, 1992). Studies in lymphomas have also demonstrated that PCNA immunostaining may be of use as a marker of proliferative activity, with some prognostic significance (Kamel *et al.*, 1991; Woods *et al.*, 1991). In 194 patients with stage III carcinoma of the cervix treated with radiation therapy alone a strong correlation was found between the PC10 index and prognosis, suggesting its potential as a prognostic indicator in patients with advanced cancer to be treated with this modality (Oka *et al.*, 1992).

The vast majority of patients with ovarian cancer present with advanced-stage disease. Adequate debulking surgery is of prognostic importance and, although the disease is chemoresponsive, relapse is often inevitable with a 5 year survival rate in stage III disease of about 20%, virtually unchanged in the last few decades (American Cancer Society, 1986). This study shows that there is a trend towards high PC10 values in patients with poor survival and, importantly, in those patients who had undergone optimal debulking surgery (<2 cm residual disease), it selects out a subgroup who have double the median survival (44 as compared with 22 months). This suggests the need for a prospective randomised trial to establish the clinical significance of this prognostic marker. A knowledge of the proliferative activity of tumours may be useful in the evaluation of prognosis and provide a means of selecting patients for appropriate therapy. Ultimately it may be appropriate to select patients for more intensive treatment regimens – for instance maintenance cytokine therapy or radiolabelled monoclonal antibody therapy – on the basis of the biology and malignant potential of disease as determined by quantitation of proliferation, measured in this way.

**Acknowledgements**
We would like to thank Dr John Pryce-Davies for reviewing the histology on these patients, Mrs Pam Davis for contacting the referring centres and Mrs Ann Nelstrop for data analysis. This study was supported by a grant from Hammersmith and Acton Special Trustees and Hammersmith and Queen Charlotte's Special Health Authority.

**References**

ALLEGRANZA A, GIRLANDO S, ARRIGONI GL, VERONESE S, MAURI FA, GAMBACORTA M, POLLO B, DALL-PALMA P AND BARBARESCHI M. (1991). Proliferating cell nuclear antigen expression in central nervous system neoplasms. *Virchows Arch. A. Pathol. Anat. Histopathol.*, **419**, 417–423.

AMERICAN CANCER SOCIETY. (1986). *Cancer Facts and Figures*. American Cancer Society: New York.

AVANZI GC, PORCU P, BRIZZI MF, GHIGO D, BOSIA A AND PEGORARO L. (1991). Interleukin-3-dependent proliferation of the human MO-7e cell line is supported by discrete activation of late G1 genes. *Cancer Res.*, **51**, 1741–1743.

BARNABEI VM, MILLER DS, BAUER KD, MURAD TM, RADEMAKER AW AND LURAIN JR. (1990). Flow cytometric evaluation of epithelial ovarian cancer. *Am. J. Obstet. Gynecol.*, **162**, 1584–1590.

BRAVO R AND CELIS JE. (1980). A search for differential polypeptide synthesis throughout the cell cycle of He La cells. *J. Cell Biol.*, **84**, 795–802.

BRAVO R, FRANK R, BLUNDELL PA AND MACDONALD-BRAVO H. (1987). Cyclin/PCNA is the auxiliary protein of DNA polymerase delta. *Nature*, **326**, 515–517.

BRESCIANI F. (1965). A comparison of the generative cycle in normal hyperplastic and neoplastic mammary gland of the C3H mouse. In *Cellular Radiation Biology*, pp. 547–557. Williams and Wilkins: Baltimore.

BURSTIN SJ, MEISS HK AND BASILICO C. (1974). A temperature sensitive cell cycle mutant of the BHK cell line. *J. Cell. Physiol.*, **84**, 397–408.

DAWSON AE, NORTON JA AND WEINBERG DS. (1990). Comparative assessment of proliferation and DNA content in breast carcinoma by image analysis and flow cytometry. *Am. J. Pathol.*, **136**, 1115–1124.

DE CAPRIO JA, LUDLOW JW, LYNCH D, FURUKAWA Y, GRIFFIN J, PIWNICA-WORMS H, HUANG CM AND LIVINGSTON DM. (1989). The product of the retinoblastoma susceptibility gene has properties of a cell cycle regulatory element. *Cell*, **58**, 1085.

DECKER DG, MUSSEY E AND WILLIAMS TJ. (1972). Grading of gynecologic malignancy: epithelial ovarian cancer. In *Proceedings of the 7th National Cancer Congress*. pp. 223–231. JB Lippincott: Philadelphia.

FRANKFURT OS. (1967). Mitotic cycle and cell differentiation in squamous cell carcinomas. *Int. J. Cancer*, **2**, 304–310.

GARCIA RL, COLTRERA MD AND GOWN AM. (1989). Analysis of proliferative grade using anti-PCNA/cyclin monoclonal antibodies in fixed, paraffin-embedded tissues. *Am. J. Pathol.*, **134**, 733–739.

HALL PA, LEVISON DA, WOODS AL, YU CC-W, KELLOCK DB, WATKINS JA, BARNES DM, GILLETT CE, CAMPLEJOHN R, DOVER R, WASEEM NH AND LANE DP. (1990). Proliferating cell nuclear antigen (PCNA) immunolocalization in paraffin sections: an index of cell proliferation with evidence of deregulated expression in some neoplasms. *J. Pathol.*, **162**, 285–294.

HOLLSTEIN M, SIDRANSKY D, VOGELSTEIN B AND HARRIS CC. (1991). p53 mutations in human cancers. *Science*, **253**, 49–53.

JAIN S, FILIPE MI, HALL PA, WASEEM NH, LANE DP AND LEVISON DA. (1991). Prognostic value of PCNA in gastric carcinoma. *J. Clin. Pathol.*, **44**, 655–659.

JASKULSKI D, DE RIEL JK, MERCER WE, CALBRETTA B AND BASERGA R. (1988). Inhibition of cellular proliferation by antisense oligonucleotides to PCNA cyclin. *Science*, **240**, 1544–1546.

KALLIONIEMI OP, PUNNONEN R, MATTILA J, LEHTINEN M AND KOIVULA T. (1988). Prognostic significance of DNA index, multiploidy and S-phase fraction in ovarian cancer. *Cancer*, **61**, 334–339.

KAMEL OW, LE BRUN DP, DAVIS RE, BERRY GJ AND WARNKE RA. (1991). Growth fraction estimation of malignant lymphomas in formalin-fixed paraffin-embedded tissue using anti-PCNA cyclin 19A2. Correlation with Ki67 labelling. *Am. J. Pathol.*, **138**, 1471–1477.

KAPLAN EL AND MEIER P. (1958). Nonparametric estimation from incomplete observations. *J. Am. Stat. Assoc.*, **53**, 457–481.

LAMBERT HE, RUSTIN GJS, GREGORY WM AND NELSTROP AE. (1993). A randomised trial comparing single agent carboplatin followed by radiotherapy for advanced ovarian cancer: a North Thames Ovary Group Study. *J. Clin. Oncol.*, **11**, 440–448.

NURSE P. (1992). Eukaryotic cell cycle control. *Biochemical Society Transactions*, **20**, 239–242.

LEVINE AJ, MOMAND J AND FINLAY C. (1991). The p53 tumour suppressor gene. *Nature*, **351**, 453–456.

LIPPONEN PK AND ESKELIN MJ. (1992). Cell proliferation of transitional cell bladder tumours determined by PCNA/cyclin immunostaining and its prognostic value. *Br. J. Cancer*, **66**, 171–176.

LIU YC, MARRACCINO RL, KENG PC, BAMBARA RA, LORD EM, CHOU WG AND ZAIN SB. (1989). Requirement for proliferating cell nuclear antigen expression during stages of the Chinese hamster ovary cell cycle. *Biochemistry*, **28**, 2967–2974.

LOUIS DN, EDGERTON S, THOR AD AND HEDLEY-WHYTE ET. (1991). Proliferating cell nuclear antigen and Ki-67 immmunohistochemistry in brain tumors: a comparative study. *Acta Neuropathol. Berl.*, **81**, 675–679.

MERCER WE, SHIELDS MT, AMIN M, SAUVE GJ, APPELLLA E, ROMANO JW AND ULLRICH SJ. (1990). Negative growth regulation in glioblastoma tumor cell line that conditionally expresses human wild-type p53. *Proc. Natl Acad. Sci. USA*, **87**, 6166–6170.

MERCER WE, SHIELDS MT, LIN D, APPELLA E AND ULLRICH SJ. (1991). Growth suppression induced by wild-type p53 protein is accompanied by selective down-regulation of PCNA expression. *Proc. Natl Acad. Sci. USA*, **88**, 1958–1962.

MIYACHI K, FRITZLER M AND TAN EM. (1978). Autoantibody to a nuclear antigen in proliferating cells. *J. Immunol.*, **121**, 2228–2234.

OKA K. HOSHI T AND ARAI T. (1992). Prognostic significance of the PC10 index as a prospective assay for cervical cancer treated with radiation therapy alone. *Cancer*. **70**, 1545–1550.

PIGNATELLI M. STAMP GW. KAFIRI G. LANE D AND BODMER WF. (1992). Over-expression of p53 nuclear oncoprotein in colorectal adenomas. *Int. J. Cancer*. **50**, 683–688.

PREHLICH G. KOSTURA M. MARSHAK KDR. MATTHEWS MB AND STILLMAN B. (1987). The cell-cycle regulated proliferating cell nuclear antigen is required for SV40 DNA replication *in vitro*. *Nature*. **326**, 471–475.

ROSA JC. MENDES R. FILIPE MI AND MORRIS RW. (1992). Measurement of cell proliferation in gastric carcinoma: comparative analysis of Ki-67 and proliferative cell nuclear antigen (PCNA). *Histochemical Journal*. **24**, 93–101.

RUTGERS DH. WILS IS. SCHAAP AH AND VAN LINDERT AC. (1987). DNA flow cytometry, histological grade, stage and age as prognostic factors in human epithelial ovarian carcinomas. *Pathol. Res. Pract.*. **182**, 207–213.

SARRAF CE. MCCORMICK CSF. BROWN GR. PRICE YE. HALL PA. LANE DP AND ALISON MR. (1991). Proliferating cell nuclear antigen immunolocalisation in gastro-intestinal epithelia. *Digestion*. **50**, 85–91.

TALAVERA A AND BASILICO C. (1977). Temperature-sensitive mutants of BHK cells affected in the cell-cylce progression. *J. Cell. Physiol.*. **92**, 425–436.

TUBIANA M AND COURDI A. (1989). Cell proliferation kinetics in human solid tumors: relation to probability of dissemination and long-term survival. *Radiotherapy and Oncology*. **15**, 1–18.

VAN DIERENDOCK JH. WIJSMAN JH. KEIJZER R. VAN DE VELDE CJ AND CORNELISSSE CJ. (1991). Cell-cycle related staining patterns of anti-proliferating cell nuclear antigen monoclonal antibodies. Comparison with BrdUrd labeling and Ki-67 staining. *Am. J. Pathol.*. **138**, 1165–1172.

VISAKORPI T. KALLIONIEMI OP. KEIKKINEN A. KOIVULA T AND ISOLA J. (1992). Small subgroup of aggressive, highly proliferative prostatic carcinomas as defined by p53 accumulation. *J. Natl Cancer Inst.*. **84**, 883–887.

VOLM M. BRUGGERMANN A. GUNTHER M. KLEINE W. PFLEIDERER A AND VOGT-SCHADEN M. (1985). Prognostic relevance of ploidy, proliferation and resistance-predictive tests in ovarian carcinoma. *Cancer Res.*. **45**, 5180–5185.

WASSEEM NH AND LANE DP. (1990). Monoclonal antibody analysis of the proliferating cell nuclear antigen (PCNA): structural conversation and detection of a nucleolar form. *J. Cell Science*. **96**, 121–129.

WOOD AL. HALL PA. SHEPHERD NA. HANBY AM. WASEEM NH. LANE DP AND LEVISON DA. (1991). The assessment of proliferating cell nuclear antigen (PCNA) immunostaining in primary gastrointestinal lymphomas and its relationship to histological grade, S + G2 + M phase fraction (flow cytometric analysis) and prognosis. *Histopathology*. **19**, 21–27.